# 

**Published:** 1902-04

**Authors:** 


					﻿C3
Oh.
LM
QC
Q_
MJ
CS
*C
I—
GO
CO
o_
1
o
<
u
o
10
c\i
40
O
2
o’
o
G>
0)
0)
i
(0
0)
I
b-
0)
I
(0
0)
00
V“
u_
a
GO
LU
Q_
co
C_3
□
=5
O
QQ
LEADS VETERINARY JOURNALISM IN AMERICA.
Vol. XXIII.
APRIL, 1902.
No. 4.
PUBLISHED MONTHLY AT $3 A YEAR. SINGLE COPIES, 30 CENTS.
FOREIGN SUBSCRIPTIONS $3-50 PER YEAR.
THE JOURNAL
OF
Comparative Medicine
AND
VETERINARY ARCHIVES
EDITED AND PUBLISHED BY
W. HORACE HOSKINS, D.V.S.,
00
o
c
z
□
o
o
2
m
0)
o
"n
o
□0
rn
>
a
<
x
O
x
a
rn
r
<
Fl
X
<
ALL COMMUNICATIONS AND PAYMENTS SHOULD BE ADDRESSED TO
Dr. W. Horace Hoskins, Managing Editor, 3452 Ludlow St., Philadelphia, Pa.
Copies may be had on order of all news-dealers at home and abroad.
AN ADVERTISING MEDIUM UNEQUALLED IN THE VETERINARY FIELD
				

